# 

**Published:** 1849-11

**Authors:** 


### NOTICE TO READERS AND CORRESPONDENTS.

We have received Original Communications for the Journal from Drs. Thompson, Logan, Freer, Stone, Sedgwick and Brackett.

We have received, through the house of S. C. Griggs & Co., from Messrs. Lea & Blanchard, a copy of West on the Diseases of Children.

We are highly gratified to find original communications taken from our pages by onr contemporaries, but hold it to be common justice that our Journal should be credited for such articles. This remark is suggested by observing in the last No. of the Ohio Medical and Surgical Journal, three of our articles, including our editorial remarks, without a note or a mark to show that they were not written expressly for that Periodical.

### RECEIPTS FOR SEPTEMBER AND OCTOBER 1849.

| | |
|---|---|
| **INDIANA.** | |
| Drs. E. Clifford, | $2 00 |
| W. Bamford, | 2 00 |
| J. Q. A. Banta, | 2 00 |
| S. V. Miller, | 2 00 |
| C. Brackett, | 3 00 |
| **ILLINOIS.** | |
| Drs. J. Riches, | $3 00 |
| D. Ransom, | 4 00 |
| S. C. Gooding, | 3 00 |
| R. S. Maloney, | 6 00 |
| J. W. Green, | 1 00 |
| Hale, | 4 00 |
| V. C. M'Clure, | 4 00 |
| A. E. Ames, | 2 00 |
| W. Robinson, | 4 00 |
| J. H. Foster, | 5 00 |
| C. H. Morton, | 2 00 |
| C. Hansford, | 2 00 |
| C. W. Knott, | 2 00 |
| L. F. Avery, | 1 00 |
| W. W. Sedgwick, | 2 00 |
| J. Blount, | 2 00 |
| H. Douglass, | 2 00 |
| Townsend Seeley, | 4 00 |
| Jas. J. Beatty, | 4 00 |
| R. R. Stone, | 3 00 |
| W. J. Paugh, | 4 00 |
| J. W. Spalding, | 2 00 |
| Drs. W. W. Cavarly, | 2 00 |
| J. W. Johnson, | 4 00 |
| Geo. S. Wheeler, | 2 00 |
| A. B. Kile, | 1 00 |
| E. S. Peabody, | 2 00 |
| **IOWA.** | |
| Drs. J. M. Williams, | 1 00 |
| N. G. Sales, | 2 00 |
| B. F. Dewey, | 2 00 |
| W. L. Whitman, | 4 00 |
| J. Doror, | 2 00 |
| L. W. Hickox, | 2 00 |
| J. L. Anderson, | 2 00 |
| Dorastus Peck, | 2 00 |
| **WISCONSIN.** | |
| Drs. Arch. Sampson, | $3 00 |
| C. A. Mills, | 2 00 |
| J. L. Cummings, | 6 00 |
| N. L. Eastman, | 4 00 |
| A. & E. N. Clarke, | 4 00 |
| **MICHIGAN.** | |
| Drs. Henry Knapp, | $2 00 |
| J. Babcock, | 2 00 |
| W. W. Goff, | 1 00 |
| N. B. Welper, | 2 00 |
| Dr. Van Nus, | 2 00 |
| **OHIO.** | |
| Dr. Geo. Watt, | 2 00 |

### TO SUBSCRIBERS.

☞ The large amount of unsettled accounts on our books, and the demands of the publishing department against the Journal, very forcibly remind us of the necessity of calling upon those of our subscribers who are in arrears to remit the amounts due.

We hope it will be remembered that two dollars will pay for this volume now, but that it will take *three* after the year expires.

